# Complete mitochondrial genome of *Coregonus chadary* Dybowski

**DOI:** 10.1080/23802359.2016.1186507

**Published:** 2016-07-08

**Authors:** Shu-Qun Xue, Ying Han

**Affiliations:** Aquaculture Department, College of Animal Science and Technology, Northeast Agricultural University, Harbin, China

**Keywords:** *Coregonus chadary *Dybowski, mitochondrial DNA, mitochondrial genome

## Abstract

The complete mitochondrial genome of *Coregonus chadary* Dybowski was determined in this study. The mitogenome is 16,739bp in length and contains 1D-loop region, 2 ribosomal RNA genes, 22 transfer RNA genes and 13 protein-coding genes. The overall base composition of the heavy strand is 26.82% for A, 29.38% for C, 18.13% for G and 25.68% for T. The percentage of G + C content is 47.51%. This study for the first time presents the mitochondrial genome sequencing for *Coregonus chadary* Dybowski.

*Coregonus chadary* Dybowski belongs to Order Salmoniformes, Family Salmonidae, Subfamily Coregoninae and Genus *Coregonus*, inhabits the Amur River and Wusuli River, is a species of freshwater whitefish in the Salmonidae family (Douglas & Brunner 2002). However, its wild population has declined rapidly because of excess fishing and habitat destruction.

In this study, one adult was used for the complete mitochondrial DNA sequencing, which was collected from the Amur in Fuyuan region (Longitude 133° 40′ 08″–135° 5′20″, north latitude 47° 25′30″–48° 27′40″). The specimen is stored in Heilongjiang River Fisheries Research Institute and its accession number is HLJFY107. The total genomic DNA was extracted from alcohol-preserved fin using the traditional phenol–chloroform method (Taggart JB et al. 1992). The sequencing results were then assembled using ContigExpess 9.0 software (http://www.megasoftware.net/megamac.php). The location of protein-coding genes was determined by comparing it with the corresponding known sequences of other *Coregonus* fish species. Also, the transfer RNA (tRNA) genes were identified using the program tRNAscan-SE 1.21 (http://lowelab.ucsc.edu/tRNAscan-SE). The phylogenetic trees were identified using the program MEGA 6.06.

The complete mitochondrial genome length of *Coregonus chadary* Dybowski was 16,739 base pairs in length (GenBank accession number KU530206). It consisted of 13 protein-coding genes, 2 rRNA genes, 22 tRNA genes and 1D-loop region. The overall base composition of the heavy strand is 26.82% for A, 29.38% for C, 18.13% for G and 25.68% for T. The percentage of G + C content is 47.51%. To investigate the divergence of the representative Salmonidae fish accessions, we constructed phylogenetic trees on the basis of the sequence variations. As shown in maximum-lLikelihood tree (Figure 1), our sequence was clustered in genus *Coregonus*, which included *Coregonus nasus, Coregonus clupeaformis, Coregonus cluncaformis, Coregonus muksum* and *Coregonus oxyrinchus.*

**Figure 1.**
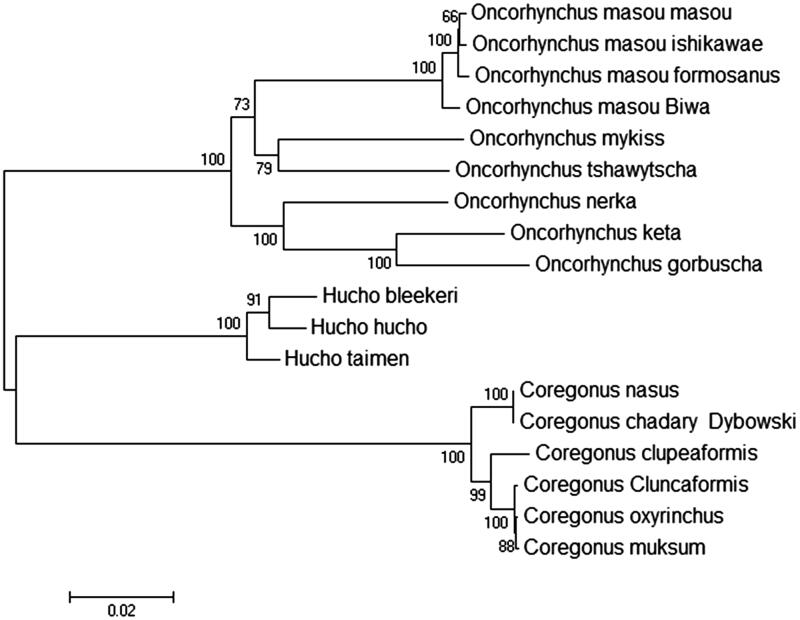
Genetic analysis of 18 Salmonidae fish strains using mitochondrial genome resequencing data. Data sources: *Oncorhynchus masou masou* (DQ864465.1); *Oncorhynchus masou ishikawae* (NC_008746.1); *Oncorhynchus masou formosanus* (NC_008745.1); *Oncorhynchus masou* 'Biwa' (NC_009262.1); *Oncorhynchus mykiss* (KP085590.1); *Oncorhynchus tshawytscha* (NC_002980.1); *Oncorhynchus nerka* (EF055889.1); *Oncorhynchus keta* (NC_017838.1); *Oncorhynchus gorbuscha* (EF455489.1); *Hucho bleekeri* (KF908853.1); *Hucho hucho* (NC_025589.1); *Hucho taimen* (NC_016426.1); *Coregonus nasus* (NC_020760.1); *Coregonus clupeaformis* (NC_020762.1); *Coregonus cluncaformis* (KT375339); *Coregonus oxyrinchus* (JQ661417.1); *Coregonus muksum* (NC_028593.1).

## References

[CIT0001] Douglas MR, Brunner PC 2002 Biodiversity of central Alpine Coregonus (Salmoniformes): impact of one-hundred years of management. Ecol Appl. 12:154–172.

[CIT0002] Taggart JB, Hynes RA, Prodoh PA, Ferguson A 1992 A simplified protocol for routine total DNA isolation from salmonid fishes. J Fish Biol. 40:963–965.

